# Ovarian Sertoli-Leydig Cell Tumors in a Three-Year-Old Child

**DOI:** 10.7759/cureus.63554

**Published:** 2024-07-01

**Authors:** Hatim Jabri, Fatoumata Binta Balde, Mohammed Mahmoud, Othmane Alaoui, Abdelhalim Mahmoudi, Khalid Khatalla, Youssef Bouabdallah

**Affiliations:** 1 Pediatric Surgery, Hassan II Hospital of Fez, Sidi Mohamed Ben Abdellah University, Faculty of Medicine and Pharmacy of Fez, Fez, MAR

**Keywords:** salphingo-oophorectomy, virilization, child, ovarian neoplasm, sertoli-leydig cell tumors

## Abstract

Ovarian Sertoli-Leydig cell tumors (SLCT) are extremely rare malignant tumors deriving from the sex cord stroma. An abdominal mass and a virilization syndrome dominate the clinical symptoms. This particular tumor poses diagnostic and therapeutic problems. Prognosis depends on staging (the International Federation of Gynecology and Obstetrics (FIGO)/tumor, node, metastasis (TNM)) and differentiation. The treatment is surgical, combined with adjuvant chemotherapy in certain cases.

We report the case of a three-year-old girl admitted to our department for signs of virilization with an abdominal mass. The literature does not contain any reports of a younger case. Ovarian SLCTs should be considered in every girl presenting with signs of virilization and a lower abdominal mass. The prognosis and management depend on the results of the histological analysis and extension evaluation in order to define therapeutic management.

## Introduction

Ovarian tumors are rare in children [1]. Sertoli-Leydig cell tumors (SLCTs) represent less than 0.5% of all primary ovarian tumors [2,3]. Common symptoms seen in patients include virilization symptoms and abdominal mass. At the time of diagnosis, almost all cases (97%) are unilateral, and 80% are limited to the ovary [3,4]. The prognosis is good and depends on the stage and degree of differentiation [4]. The treatment remains challenging and is based on surgery and adjuvant chemotherapy [5]. There is a lack of standardized protocols for patient treatment since each center follows its own approach. Here, we present a case of poorly differentiated SLCT in a three-year-old girl.

## Case presentation

**Figure 1:**
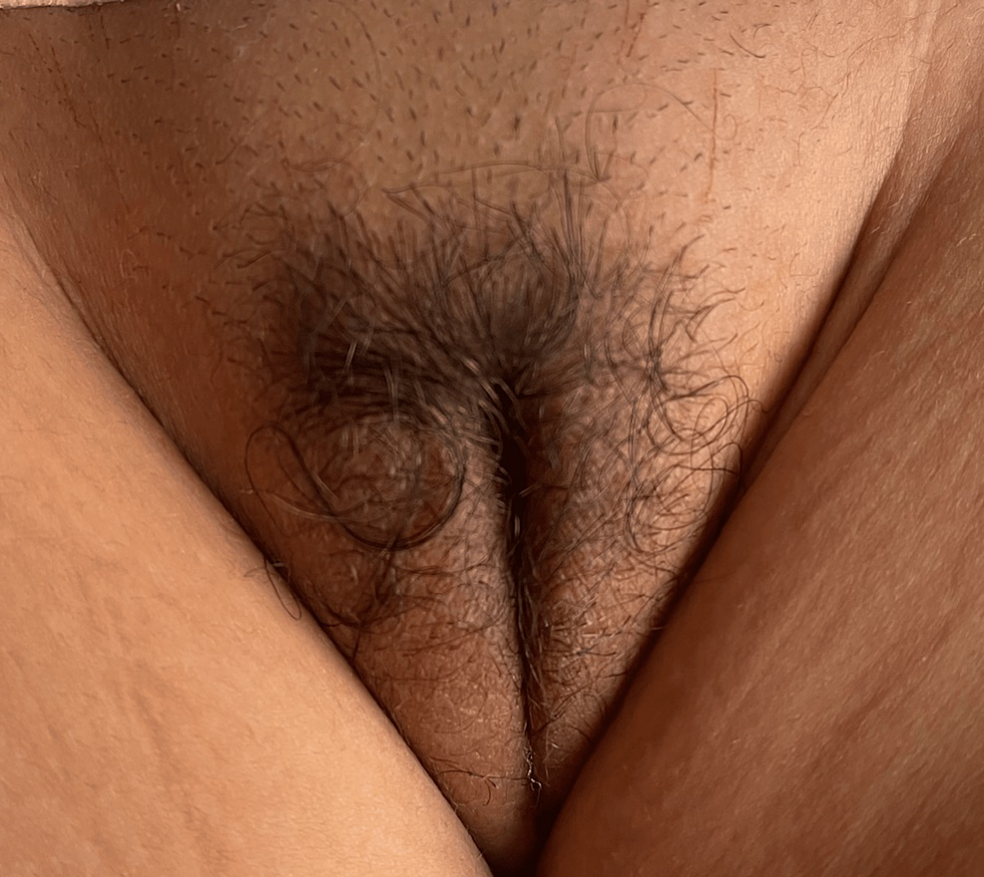
A clinical image showing pubic hair in a three-year-old girl child

**Figure 2:**
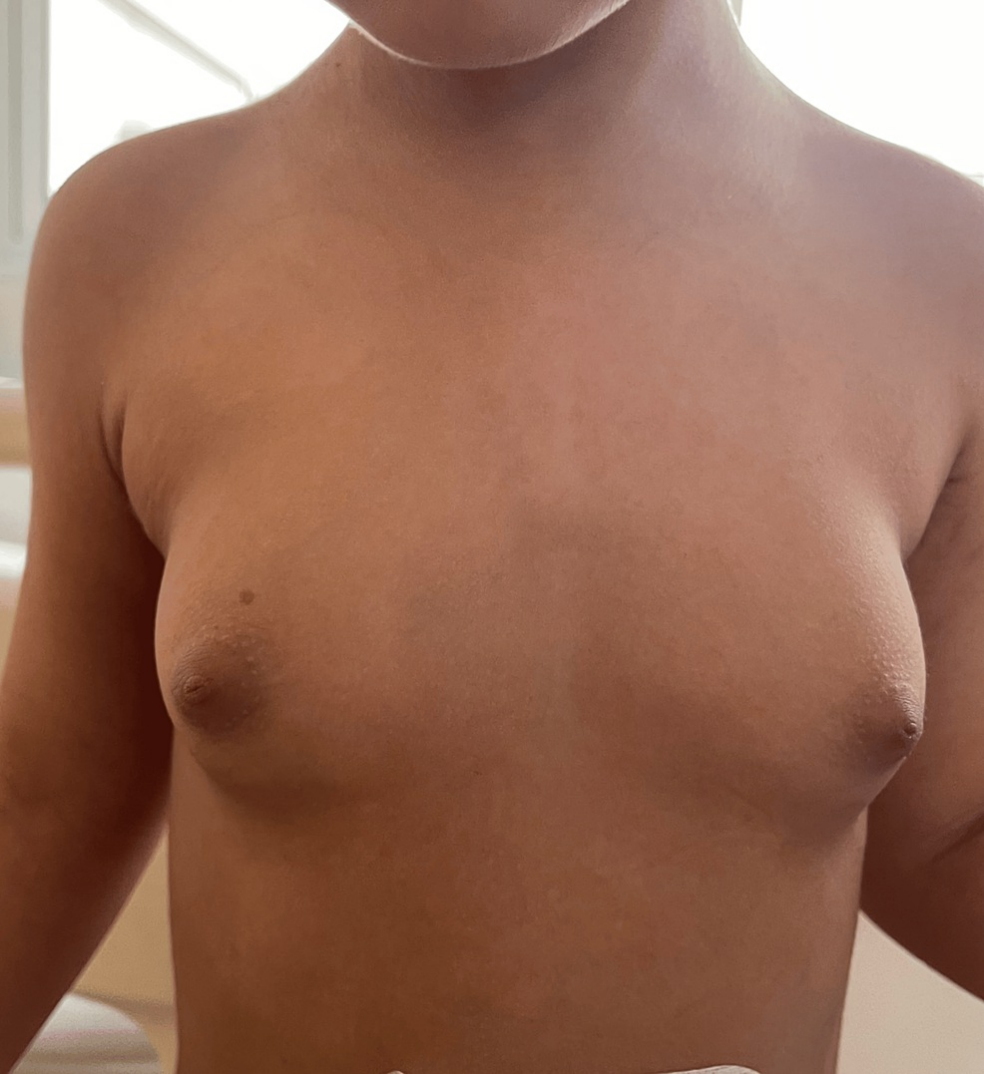
A clinical image showing breast development in a three-year-old girl child

A three-year-old girl child with no significant medical history was presented to the pediatric department for pubic and axillary hair growth, accompanied by an increase in mammary gland size. On general examination, the patient was hemodynamically and respiratory stable, and apyretic, with the presence of a hard hypogastric mass measuring 10 cm in length, not painful to palpation, and fixed to the superficial and deep planes. The patient also had pubic hair (Figure 1) and was in stage 3 of breast development (Figure 2).

**Figure 3:**
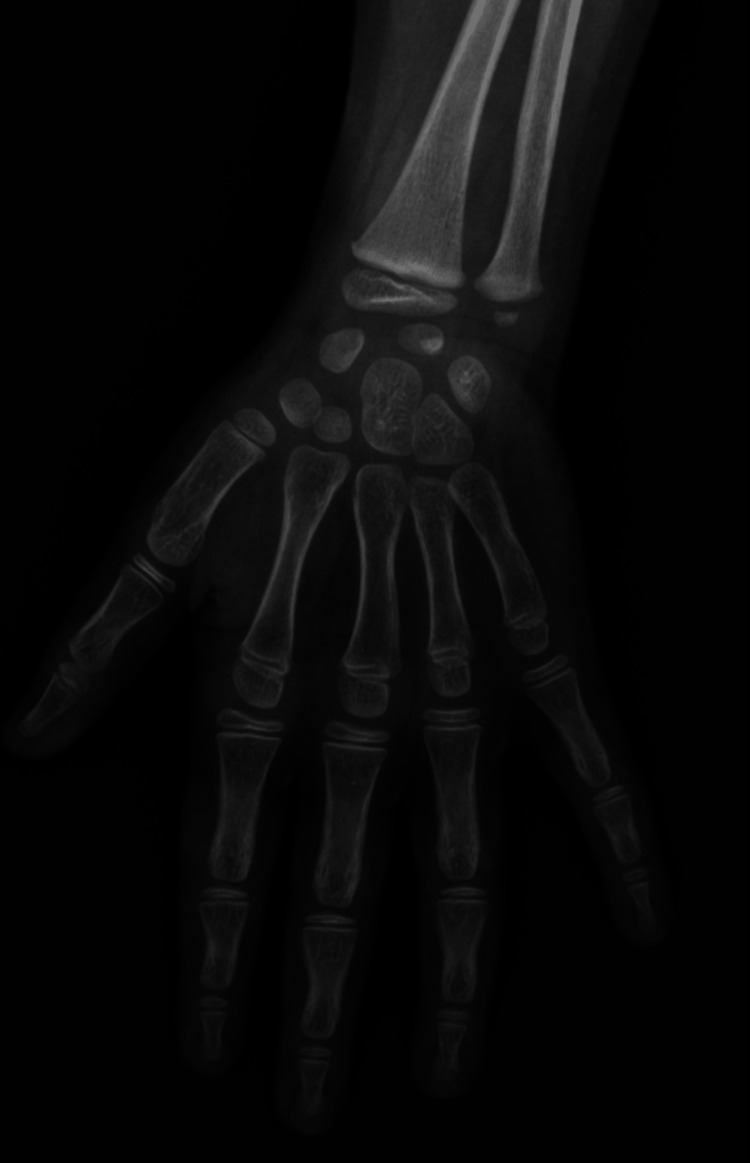
The hand and wrist x-rays of our three-year-old patient demonstrated her bone age at six years.

Her weight was 21 kilograms (95^th^ percentile), and her height was 106 cm (95^th^ percentile). We estimated the bone age at six years (advanced by three years) (Figure 3).

**Figure 4:**
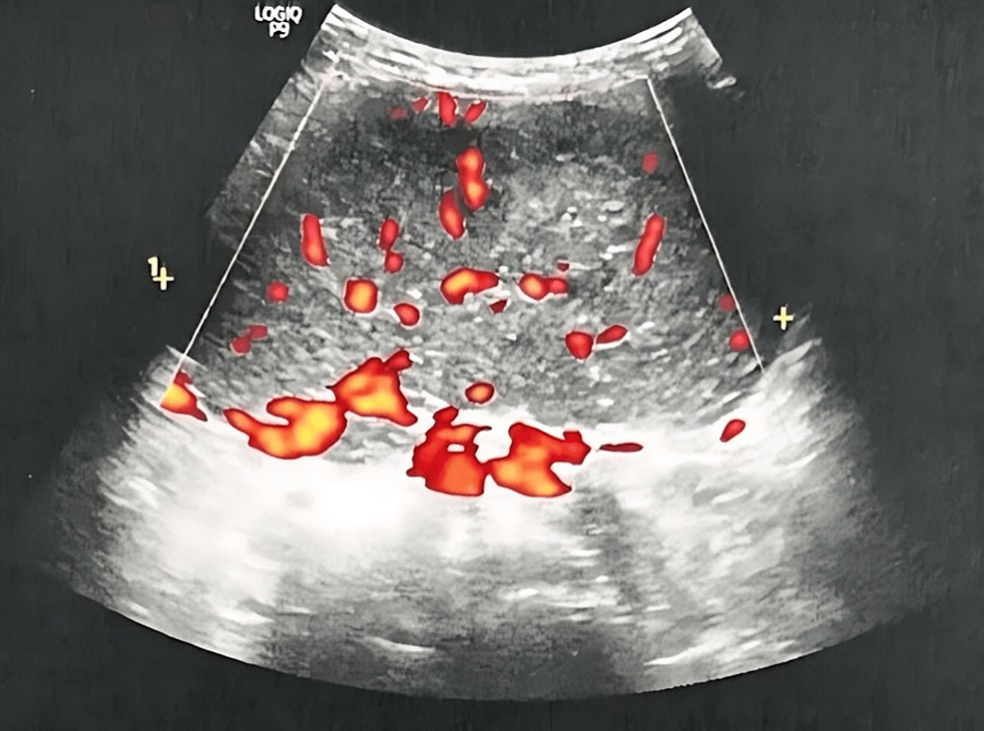
Pelvic ultrasound revealed an 11 x 6 cm median solid lesion more developed on the right side.

Pelvic ultrasound revealed an 11 x 6 cm median solid lesion that was more developed on the right side, suggesting an ovarian neoplasm (Figure 4).

**Figure 5:**
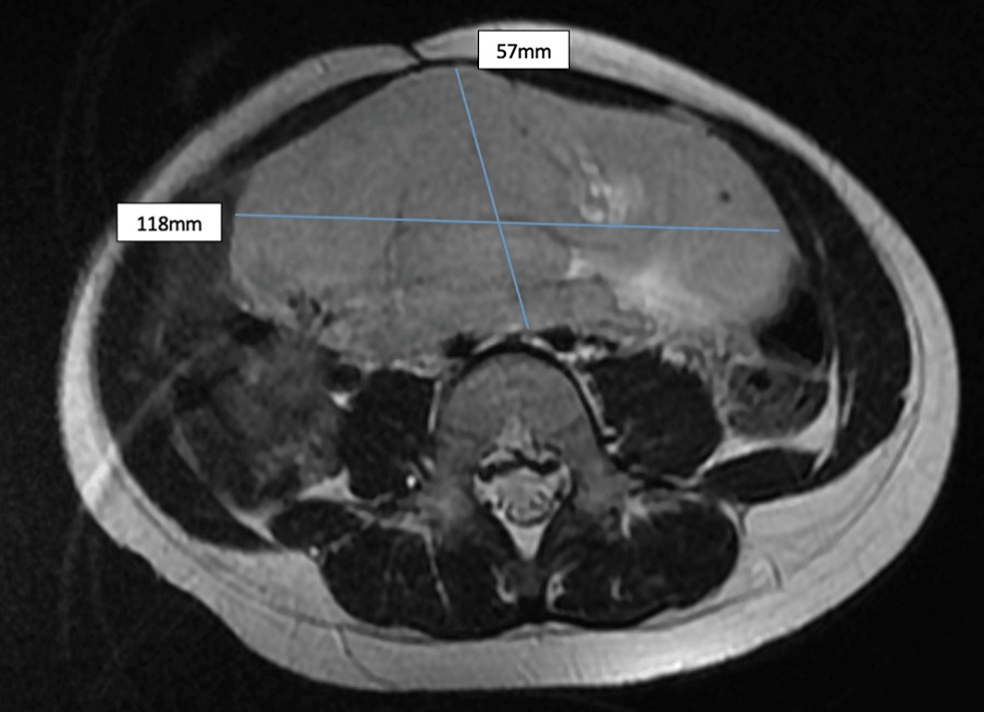
Pelvic MRI showed the presence of a voluminous right ovarian mass, lobulated in outline. The blue lines show the limits and dimensions of the tumors.

An MRI revealed the presence of a voluminous right ovarian mass, measuring 13 x 10 x 6 cm in diameter, lobulated in outline, with heterogeneous enhancement after contrast, delimiting a fleshy component and a necrotic component without peritoneal effusion or a carcinosis nodule, suggesting ovarian dysgerminoma (Figure 5).

**Figure 6:**
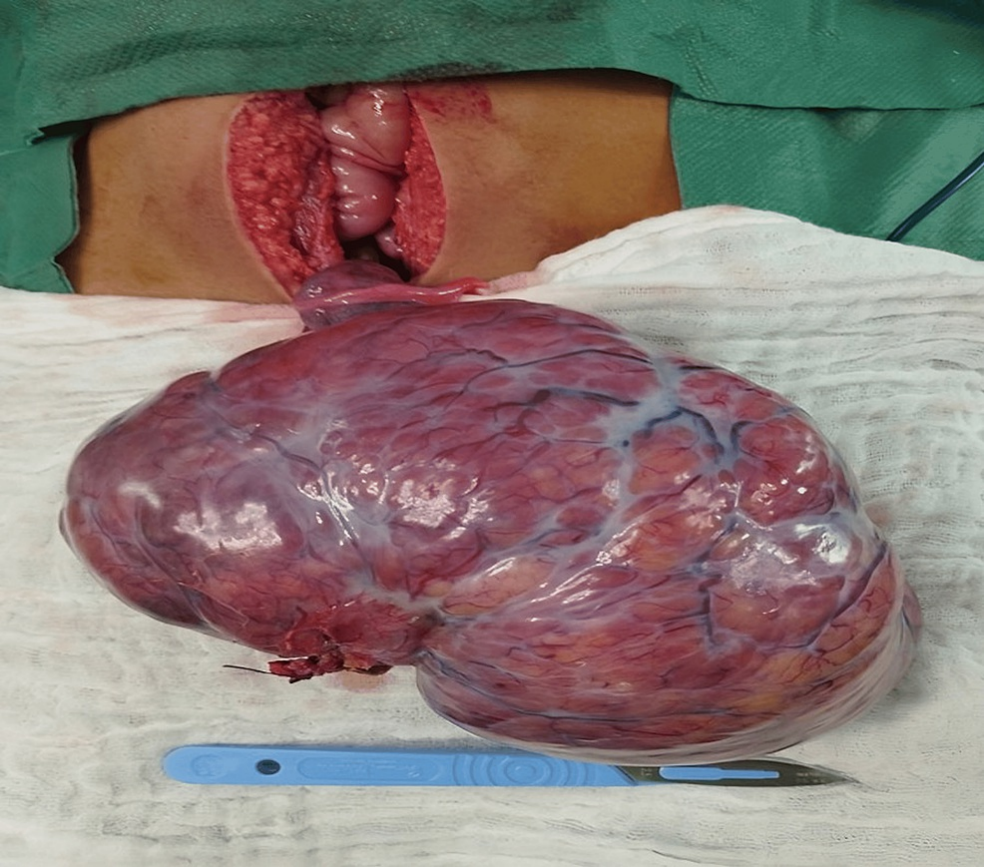
Surgical image showing the ovarian tumor

A biological check-up revealed elevated serum estradiol (82 ng/ml) and lactate dehydrogenase (LDH, 386 IU/ml) levels. However, levels of testosterone, luteinizing hormone (LH), alpha-fetoprotein, human chorionic gonadotropin (hCG), follicle-stimulating hormone (FSH), and dehydroepiandrosterone (DHEA) were normal. A multidisciplinary staff discussed the patient's case and decided to proceed with a right oophorectomy. Given the size of the tumor, open surgery was performed through a midline incision under the umbilicus. Surgical exploration revealed a large, encapsulated, and firm mass over the right ovary that was not adherent to the pelvic wall or the other organs (Figure 6). The right ovarian tube appeared to be normal, with no signs of peritoneal carcinosis. We took peritoneal fluid for a cytological study. We performed a right oophorectomy while preserving the fallopian tube stump.

**Figure 7:**
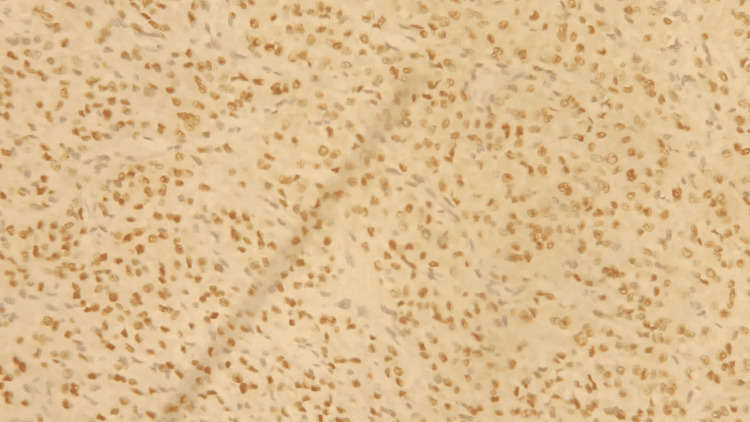
Molecular study showed expression of androgen receptors

The immediate postoperative course was straightforward, leading to the patient's discharge from the hospital on the second day. The anatomopathological study showed an undifferentiated tumor of the sex cords and ovarian stroma classified as a Sertoli-Leydig tumor (Figure 7). The molecular study found that tumor cells diffusely expressed vimentin and locally expressed CK, calretinin, inhibin, CD99, and androgen receptors. There were no cancerous cells detected in the peritoneal cytology.

**Figure 8:**
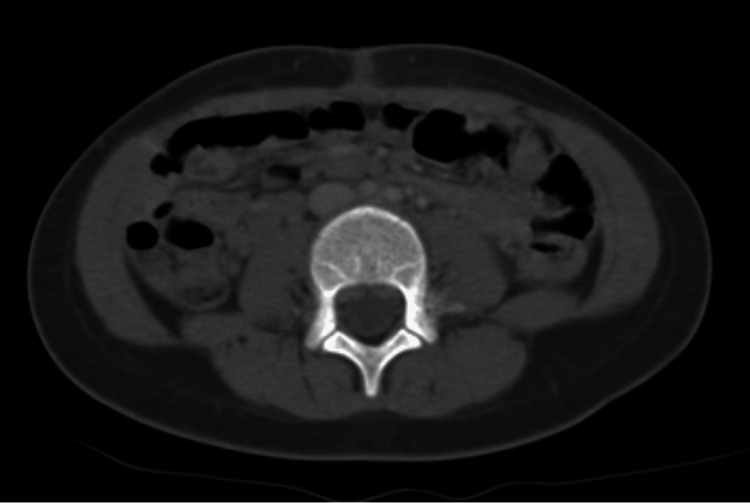
Pelvic scan one year after surgery showed no signs of recurrence.

The tumor was classified as stage IA as per the International Federation of Gynecology and Obstetrics (FIGO) classification, but given the undifferentiated character of the tumor, it was decided to proceed with adjuvant chemotherapy using the TGM 95 protocol (etoposide, ifosfamide, and cisplatin (VIP) cure). The patient received two VIP treatments. Radiological (Figure 8) and hormonal assessments were performed and came back normal.

After one year, we noted a regression of the signs of virilization. We decided to conduct regular control every three months, including an abdominopelvic ultrasound and hormonal tests. We followed up with the patient for two years without any recurrences.

## Discussion

Ovarian tumors are rare in children [1]. The SLCT is an uncommon neoplasm classified as a sex cord-stromal cell ovarian tumor [6]. These tumors often appear in the second to fourth decade of life [5,7]. Its incidence varies between 0.1% and 0.5%, depending on the series [1,2,8]. Less than 10% of SLCTs appear before puberty [9]. In children, this type of tumor is characterized by the presence of signs of virilization associated with an abdominal mass or pain [3,10].

Most SLCTs are unilateral, confined to the ovary, and typically <5 cm in diameter [9,11]. In our case, the patient presented signs of virilization, with a pelvic mass measuring 10 cm on the long axis. The hormone test finds an elevated testosterone level in 80% of cases [6,11], In our case, the testosterone level was normal. Radiological assessment, including ultrasound, remains a useful approach to exploring abnormalities in the pelvic region without confirming the type of tumor; thus, CT, MRI, and PET scans will allow a better analysis of the tumor and its extension [5,11].

Treatment is essentially surgical, considering the stage of the tumor and the degree of differentiation. Meyer [5] identified three histological tumor categories based on differentiation: well, intermediate, and undifferentiated tumors. Typically, intermediate and poorly differentiated neoplasms are more endocrine and seen at a younger age. O'Hern and Neubecker [5] discovered a positive correlation between the occurrence of virilization and a decrease in tumor differentiation. Our patient was three years old with a poorly differentiated tumor. Therapeutic management remains challenging for the surgeon and the oncologist, given the lack of studies dealing with this pathology and the unknown potential for malignancy and evolution. Unilateral salpingo-oophorectomy is recommended if the tumor is limited to the ovary without involving the controlateral ovary [5,10].

In cases of advanced-stage metastasis or intermediate and poorly differentiated tumors, surgery with postoperative chemotherapy or radiotherapy must be performed [10,12,13]. In our case, the tumor has been classified as FIGO stage IA, but given the undifferentiated character of the tumor, adjuvant chemotherapy was performed, given that several studies have demonstrated the benefits of adjuvant therapy in preventing recurrence [14]. Different series proposed multiple chemotherapy protocols [6], and for our patient, we chose a combination of etoposide, ifosfamide, cisplatin, and mesna.

Regular follow-up is necessary due to the high risk of recurrence, especially in the first year [5,14]. Sigismondi et al. [15] found that patients with stage 1 neoplasm had survival rates of 92.3% after five years, but in the case of a moderately or undifferentiated tumor, the five-year survival rate falls to around 70%. The mortality rate in SLCTs varies from 12% to 34% [5]. The principal limitation of this case was the absence of a long-term follow-up.

## Conclusions

Sertoli-Leydig cell tumors are rare tumors and are generally benign. Most patients are diagnosed at the stage of virilization and pelvic masses. Their management is still not well codified, and treatment must be personalized according to age, stage, and degree of differentiation. Close and regular follow-up is required in order to identify any recurrences.
